# Critical insight on ‘respiratory swallow coordination training using bimodal signal biofeedback for patients with post-stroke dysphagia: a letter to editor

**DOI:** 10.1080/07853890.2026.2616884

**Published:** 2026-01-20

**Authors:** Prashant Kumar, Kanika

**Affiliations:** Maharishi Markandeshwar Institute of Physiotherapy and Rehabilitation, Maharishi Markandeshwar (Deemed to be University), Ambala, India

Dear Editor

We are writing to offer insight on the randomized controlled trial by wang et al. entitled “Respiratory swallow coordination training using bio models signal biofeedback for patients with post stroke dysphagia published in Annals of Medicine. This pioneering study employed an innovative bimodal biofeedback approach that synchronized respiratory airflow with accelerometry signals, aiming to enhance respiratory swallow coordination and improve swallowing function in individuals recovering from stroke [1].

The physiological concept underlying this work is well substantiated. Previous research has established that initiating and concluding swallowing during the expiratory phase particularly within the Exhale swallow Exhale (E-SW-E) sequence supports safer airway management and reduces aspiration risk [2]. By prioritizing coordination-based retraining over traditional strength or maneuver-oriented therapies, this study meaningfully advances current dysphagia rehabilitation frameworks [3].

Despite the methodological strength of the trial, several considerations were not attention the Functional Oral Intake Scale (FOIS) used as the primary outcome, was analyzed through separate non parametric comparison at post intervention and follow up [1]. Since FOIS represents ordinal data collected across multiple time points the application of repeated major analytical models such as mixed effect of ordinal regression analysis could have provided a more comprehensive understanding of within and between group variation.

Additionally, while the intervention group demonstrated functional gains and reduced penetration aspiration events, temporal and kinematics measures obtained *via* video fluoroscopic swallowing study (VFSS) revealed limited intergroup difference [1]. This divergence raises an important interpretive issue concerning whether improvements in respiratory swallow coordination translate into miserable biomechanical adaptations. Prior evidence suggests that functional recovery may occur even in the absence of current VFSS parameter changes. However, elucidating this relationship remains crucial to validate the mechanistic basis of coordination driving therapy [4].

Moreover, respiratory swallow coordination was quantified through the proportion of E- SW- E cycles identified in dual signal recordings [1]. Given that accelerometer based and nasal air flow derived data can be affected by motion artifacts and irregular breathing rhythms common in neurological populations including reproducibility metrics or interrater reliability estimates would enhance confidence in the accuracy and clinical interpretability of these findings [5].

Finally, the clinical implication of the coordination data could we further strengthen by exploring their association with key functional measures. Investigating correlation between shifts in E-SW-E frequency and improvements in FIOS or penetration aspiration scale score could help determine whether optimized respiratory swallow timing acts as casual mechanism of recovery or merely accompanies functional improvement.

In conclusion, the study by Wang et al. offers a valuable and forward-looking contribution to post stroke dysphagia rehabilitation. By emphasizing respiratory swallow coordination through bimodal biofeedback, it opens an important avenue for therapeutic innovation.

## Data Availability

There is no associated data with this manuscript.
